# A single cell transcriptional profile of benign prostatic hyperplasia

**DOI:** 10.1038/s41598-025-02417-w

**Published:** 2026-03-14

**Authors:** Rei Unno, Jon Akutagawa, Hanbing Song, Keliana Hui, Yih-An Chen, Julia H. Pham, Jean Lee, Heiko Yang, Franklin W. Huang, Thomas Chi

**Affiliations:** 1 Division of Hematology and Oncology, Department of Medicine, University of California San Francisco, San Francisco, USA; 2 Helen Diller Family Comprehensive Cancer Center, University of California San Francisco, San Francisco, USA; 3 Bakar Computational Health Sciences Institute, University of California San Francisco, San Francisco, USA; 4 Institute for Human Genetics, University of California San Francisco, San Francisco, USA; 5 Department of Urology, University of California San Francisco, San Francisco, USA; 6 Division of Hematology and Oncology, Department of Medicine, San Francisco Veterans Affairs Medical Center, San Francisco, USA; 7 Department of Nephro-urology, Nagoya City University Graduate School of Medical Sciences, Nagoya, Japan; 8 Biohub San Francisco, San Francisco, USA

**Keywords:** Benign prostatic hyperplasia, Lower urinary tract symptoms, Single cell RNA-sequencing, Inflammation, HoLEP, Transcriptomics, Prostate, Benign prostatic hyperplasia, Bioinformatics, Genome informatics, RNA sequencing

## Abstract

Benign prostatic hyperplasia (BPH) is characterized by excessive cell proliferation and inflammation and affects most aging men. The development of new therapies for BPH requires a deeper understanding of the underlying pathophysiology and cellular components of BPH. Single-cell RNA-sequencing was performed on prostate tissue from 15 patients undergoing holmium laser enucleation of the prostate for treatment of BPH. Clustering and differential expression analysis on aligned single-cell RNA-seq data was performed to annotate all cell types. 16,234 cells were analyzed and specific stromal, epithelial, and immune subgroups were found to be strongly associated with inflammation. A rare luminal subgroup was identified and pseudotime analysis indicated this luminal subgroup might give rise to other luminal cells. Using a gene set derived from epithelial stem cells, we found that this luminal subgroup had a significantly higher stem cell signature score than all other epithelial subgroups, suggesting this subgroup is a luminal precursor state. Ligand-receptor interactions between stromal, epithelial, and immune cells were explored with CellPhoneDB. Significant interactions involving MIF, a pro-inflammatory cytokine that promotes epithelial cell growth and inflammatory response in the prostate, were identified between the progenitor-like luminal subgroup and both fibroblasts and macrophages. Our single-cell profiling of BPH provides a roadmap for investigating inflammation-linked cell subgroups and highlights a progenitor-like luminal subgroup interacting with other cell groups via *MIF* that may contribute to the inflammation and cell proliferation phenotype associated with BPH.

**Supplementary Information:**

The online version contains supplementary material available at 10.1038/s41598-025-02417-w.

## Introduction

The prostate is a gland that produces seminal fluid in men and increases in size during aging in a disease process known as benign prostatic hyperplasia (BPH)^1–3^. An enlarged gland can obstruct the flow of urine and cause lower urinary tract symptoms (LUTS), which can significantly impact an individual’s quality of life^2–4^. BPH negatively impacts approximately 50% of men by age 60, and its prevalence increases by about 10% each subsequent decade^5,6^.

BPH is characterized by abnormal glandular and stromal proliferation, primarily in the transition zone (TZ)^7,8^. This proliferation is associated with chronic inflammation and fibrosis, which are considered hallmarks of BPH^9–11^. Macrophages and T cells have been implicated as the major inflammatory cell populations^12,13^.

Detailed cell states and interactions among these cell types are poorly understood as many previous characterizations have relied on unsorted bulk tissue samples and histological features^14,15^. How inflammation drives epithelial and stromal proliferation or vice versa remains unclear. Without a better mechanistic understanding of BPH pathophysiology, better pharmacotherapeutic strategies cannot be developed. The efficacy of current BPH medications – alpha-blockers and 5-alpha-reductase inhibitors (5-ARIs) – is limited, and a large number of patients continue to rely on surgical BPH procedures for relief^9,16^.

Single-cell RNA-sequencing (scRNA-seq) enables the characterization of individual cell populations and prediction of their interactions at the transcriptional level^17^. Recently, new prostate epithelial cell types, including hillock and club cells, have been detected in the normal and cancerous prostate using scRNA-seq^18,19^. While these studies have focused on prostate cancer, BPH, despite its prevalence, remains relatively unexplored^20–22^. In this study, we used scRNA-seq to define the cell states and interactions associated with BPH. We identified cellular subgroups in the stromal, epithelial, and immune compartments strongly linked to inflammation. In particular, we found a luminal epithelial subgroup that had macrophage migration inhibiting factor (MIF)-driven interactions with stromal and immune cells. This epithelial subgroup and *MIF* could contribute to the inflammation and cell proliferation phenotype associated with BPH.

## Results

### Single cell transcriptomic analysis identifies 14 cell types in benign prostatic hyperplasia

**Fig. 1:**
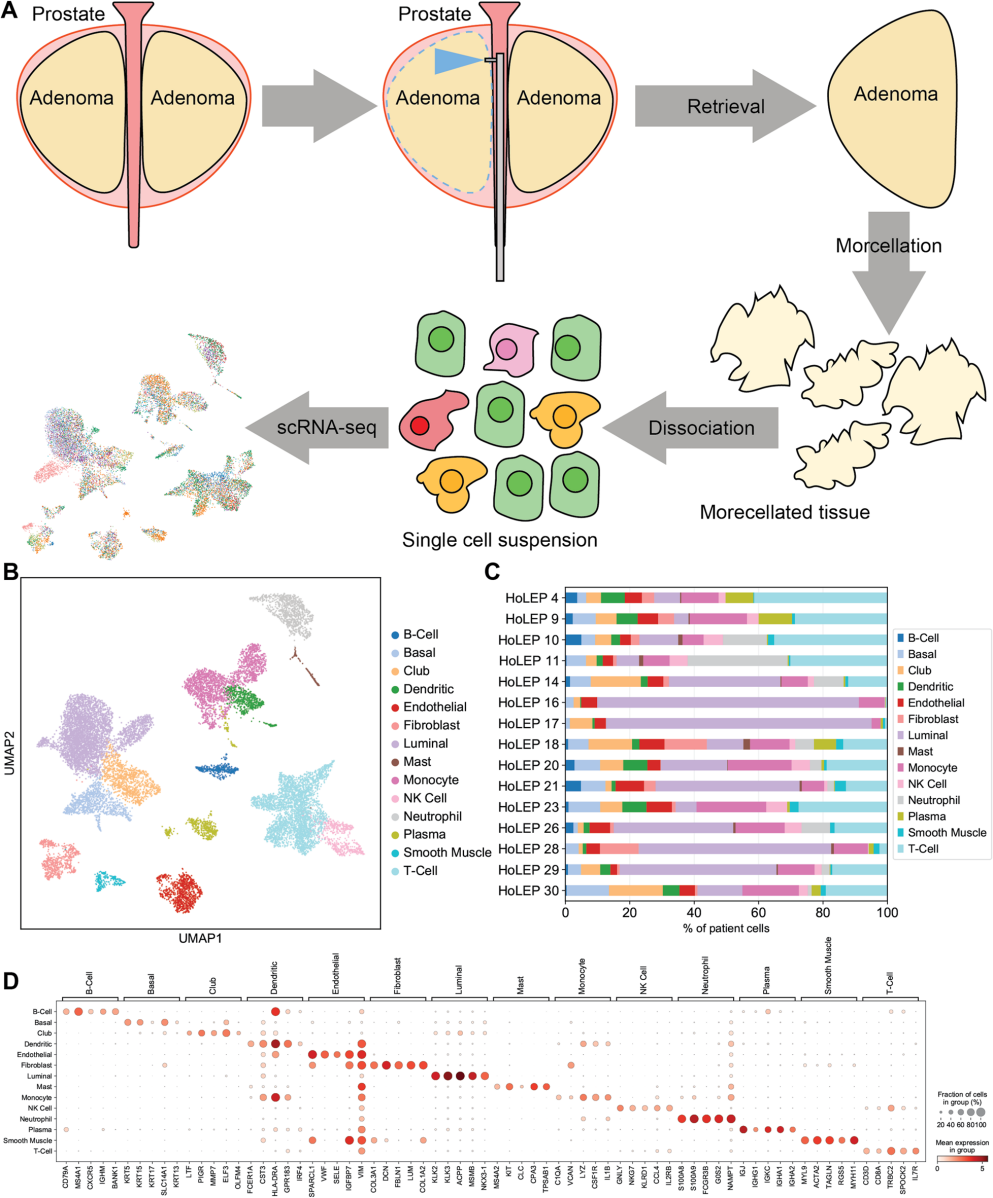
Identification of 14 cell groups in BPH samples via single cell RNA-sequencing. (**A**) Schematic of scRNA-seq of BPH cells obtained via HoLEP. (**B**) Uniform manifold approximation and projection (UMAP) of BPH transcriptomes show 14 distinct cell clusters. (**C**) Cell type composition of 15 BPH samples. (**D**) Dot plot of expression levels of known marker genes in each cell type.

Prostate tissues from BPH patients undergoing Holmium laser enucleation of the prostate (HoLEP) were dissociated into single cells and then captured and sequenced using the Seq-Well scRNA-seq platform^23,24^. After ambient RNA decontamination and removal of low-quality cells, we isolated 16,234 cells from 15 different patients (Table 1) for analysis (Fig. 1A), which was similar in number to previous studies^19^. Using a graphical clustering method, 14 clusters of distinctive transcriptomic profiles were initially identified using a semi-supervised annotation pipeline (Fig. 1B). Differentially expressed genes (DEGs) in each cluster were computed, and canonical cell-type-specific markers were used to annotate epithelial cells, endothelial cells, stromal cells, and immune cells (Fig. 1D, Supplementary material, Table S1, S2).

**Table 1:** Clinical data of BPH samples from 15 patients.

Patient	Ethnicity	Age	Size (cc)	5ARI treatment status
HoLEP 4	African American	66	118	No
HoLEP 9	African American	60	103	No
HoLEP 10	White	75	48	No
HoLEP 11	White	68	102	No
HoLEP 14	White	59	58	Yes
HoLEP 16	White	74	162	No
HoLEP 17	White	56	68	No
HoLEP 18	White	83	105	No
HoLEP 20	White	82	50	Yes
HoLEP 21	White	67	50	No
HoLEP 23	East Asian	70	170	No
HoLEP 26	White	72	116	No
HoLEP 28	White	66	50	No
HoLEP 29	East Asian	78	50	Yes
HoLEP 30	White	74	155	Yes

Epithelial cells were the most abundant population and could be separated into three major subtypes, including basal, club, and luminal epithelial cells. Basal cells were enriched in basal markers such as *KRT5*, *KRT15*, and *TP63*, and club cells were enriched with previously established club cell markers including *PIGR*, *MMP7*, *LTF*, and *SCGB3A1*. Luminal cells, identified by expression of *KLK3*, *KLK2*, and *ACPP*, were the largest epithelial population detected (*n* = 4,270 cells, 68.3% of all epithelial cells).

As with epithelial cells, immune cell analyses also revealed several subtypes. T cells that expressed *CD8A* and *CCL5* were the most abundant immune cell type present (*n* = 3,370 cells), followed by monocyte cells (*C1QA* and *IL1B*, *n* = 1,952), neutrophils (*FCGR3B*, *n* = 892), dendritic cells (*FCER1A*, *n* = 585 cells) and then plasma cells (*IGJ* and *IGHA1*, *n* = 490 cells). Stromal cells consisted of fibroblasts (*FBLN1* and *DCN*) and smooth muscle cells (*ACTA2* and *MYLA9*). Most cell types were identified across multiple patients and allowed a cell type composition comparison between different clinical features (Fig. 1C). No significant associations were found between broad cell type composition and any single clinical feature (ethnicity, patient age, prostate size, treatment status) in this cohort (Supplementary material, Fig. S3).

### Distinct fibroblast groups are associated with inflammation pathways

**Fig. 2:**
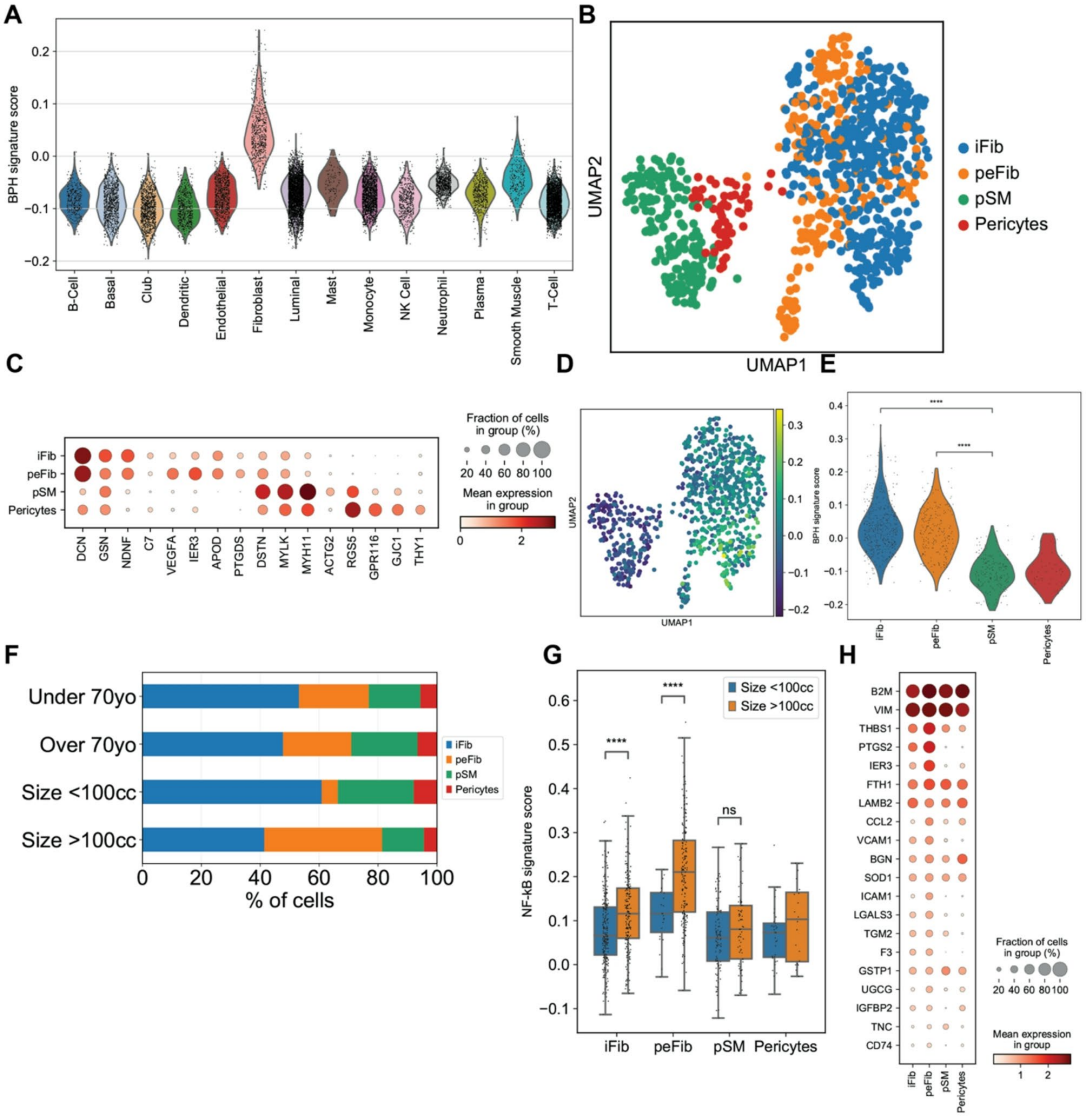
Single-cell profiling of the stromal cell compartment in BPH patients. (**A**) Violin plot of BPH signature score, derived from Middleton et al., across all cell groups. (**B**) UMAP clustering of stromal cells. (**C**) Dot plot of selected differentially expressed genes across the 4 stromal subtypes. (**D**) Feature plot of stromal cells, showing BPH-associated gene expression of each cell. (**E**) Violin plot of BPH signature score across the 4 stromal subtypes. (**F**) Stromal cell compositions of patients younger or older than 70 or with prostates smaller or larger than 100 cc. (**G**) NF-κB pathway gene signature scores of stromal cells by prostate size. (**H**) Dot plot of NF-κB pathway associated gene expression in the 4 stromal subtypes.

To test for cell types associated with BPH, we performed signature score analysis and identified fibroblasts as the cell type with the strongest enrichment of a BPH signature gene set derived from a previous bulk RNA-seq study^15^ (Fig. 2A). To further investigate this population, we clustered the stromal population into peri-epithelial fibroblasts (peFib), interstitial fibroblasts (iFib), prostate smooth muscle cells (pSM), and pericytes based on marker genes from previously annotated stromal cells^21^ (Fig. 2B, C, and Supplementary material, Fig. S4, Table S3). Canonical fibroblast markers – *LUM*, *VCAN*, and *BMP5* – were expressed in both fibroblast clusters. The smooth muscle population was enriched in smooth muscle specific markers *ACTA2*, *MYH11*, and *MYL9*^18^. The pericytes were characterized by *THY1*, *RGS5*, and *GJC1* expression. *C7*, which encodes for complement component C7, has been previously reported as a marker for interstitial fibroblasts in BPH samples^21^, was found to be expressed in a small population of iFib cells. We found that the interstitial fibroblasts were most enriched in the previously established BPH signature gene set (Fig. 2D, E) and peri-epithelial fibroblasts were the second most enriched population for the BPH signature in this cohort.

Next, we examined if these fibroblast clusters are associated with certain clinical features. We found stromal cells in 14 patients; only Patient 16 had no stromal cells detected (Supplementary material, Figure S5A). We found that the peri-epithelial fibroblast clusters consisted of cells from 11 patients, with Patients 4, 9, and 18 having the largest proportion of peri-epithelial cells. Interstitial fibroblasts were also found in 11 patients. Patient 28 had the largest proportion of interstitial fibroblasts, followed by Patient 18 and 21. Patients 17 and 20 had no fibroblasts detected. Prostate smooth muscle was detected in 14 samples and pericytes were detected in 10. We found that patients with prostates larger than 100 cc tended to have more peri-epithelial and less interstitial fibroblasts (Fig. 2F) (*p* < 1e-8, Fisher’s exact test). Expression of genes associated with the inflammatory TNF/NF-κB pathway was significantly higher in fibroblasts from patients with larger prostates (Fig. 2G) (*p* < 1e-5, Mann-Whitney U test), particularly in the peri-epithelial fibroblasts. Patients treated with 5-ARIs also had less fibroblasts (Fig. S5B). Peri-epithelial fibroblasts had the highest expression of NF-κB pathway genes (Fig. 2H, and Supplementary material, Fig. S5C, D), specifically in peFib cells that strongly expressed *TIMP1*, *VEGFA*, *IER3*, and *CCL2* (Supplementary material, Fig. S5E). Taken together, these data suggest that peri-epithelial fibroblasts in prostates with BPH exhibit an inflammatory phenotype consistent with the disease.

### A potential progenitor luminal epithelial cell subpopulation interacts with fibroblasts through MIF

**Fig. 3:**
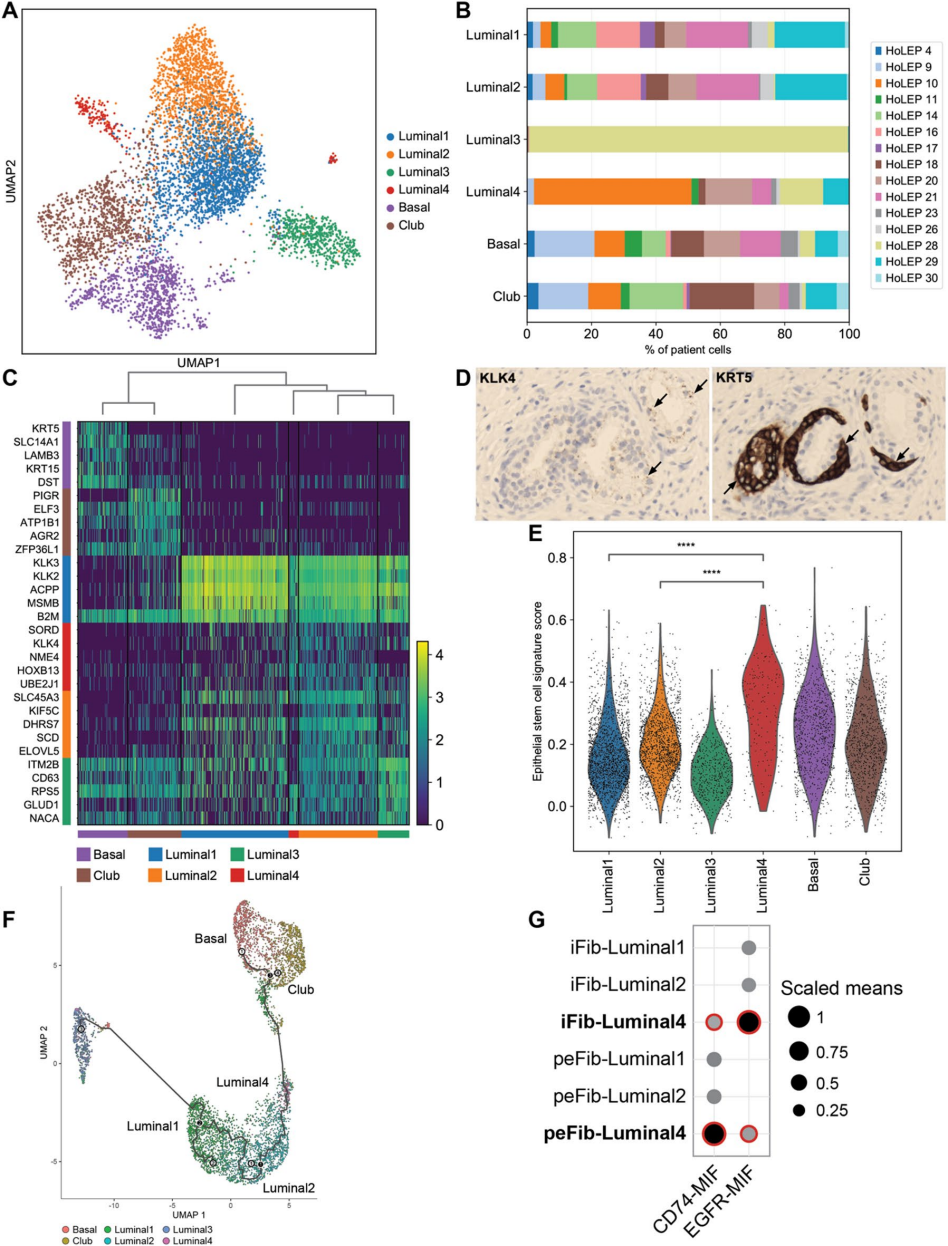
Epithelial cell characterization reveals a potential progenitor luminal subgroup. (**A**) UMAP clusters epithelial cells into 6 subgroups. (**B**) Cell contributions from each sample to the epithelial subgroups. (**C**) Heatmap of differentially expressed genes in epithelial cells. (**D**) Immunohistochemical staining for KLK4 (brown) and KRT15 (brown) in BPH tissue. Hematoxylin and eosin were used as counterstains. (**E**) Violin plot of epithelial stem cell-associated gene expression scores in the 6 epithelial subgroups. (**F**) Trajectory analysis of the 4 luminal subgroups. (**G**) Interaction analysis of *MIF* and other selected genes between fibroblast and luminal subgroups. Larger dot sizes represent higher means of average expression level of the two interacting molecules. Red highlights represent a significant interaction, with an adjusted p-value < 0.05.

As fibroblasts strongly express BPH-associated genes (Fig. 2A) and epithelial-to-stromal crosstalk is a known feature in BPH, we hypothesized that specific interactions might be present between BPH fibroblast states and surrounding epithelial cells that promote inflammation or cell proliferation^25^. To investigate this possibility, we first performed a subgrouping analysis of epithelial cells and identified subgroups in basal cells, club cells, and four subgroups of luminal epithelial cells according to both their gene expression profiles and their clustering patterns (Fig. 3A). Basal cells were found in all patients, but showed no strong differences in population size or gene expression across prostate age, size, or 5ARI treatment status (Supplementary material, Fig. S6). All four luminal subgroups shared high expression of canonical luminal epithelial markers, including *KLK3*, *KLK2*, *ACPP*, and *NKX3-1* (Fig. 3C) and genes associated with the androgen receptor pathway (supplementary material, Fig. S7A, B). These four luminal subgroups included two larger subgroups (*n* = 2,026 and 1,495) that were largely overlapping in gene expression profiles, a third subgroup (*n* = 595) found in only one patient, and a much smaller fourth subgroup (*n* = 186) that had contributions from multiple patients and was characterized by higher expression of *KLK4*, *HOXB13*, and *SORD* (Fig. 3B, C, and Supplementary material, Fig. S7D). We performed immunohistochemical staining of BPH cells and confirmed *KLK4* expression in luminal zones (Fig. 3D). Significantly upregulated genes in the “luminal 4” subgroup compared to the first two luminal subgroups also featured many ribosomal genes (Supplementary material, Fig. S7C, D), including *RPS15*, *RPL27A*, *RPS2*, *RPL7A*, and *RPLP0*. The top significantly enriched gene set from a gene ontology (GO) analysis of this subgroup was the KEGG Ribosome gene set (Supplementary material, Fig. S8A, B). The luminal 4 subgroup also had lower expression of genes in the AR pathway and higher expression of genes in the MYC pathway compared to the other luminal subgroups (Supplementary material, Fig. S8C), similarly seen in some luminal cells in aggressive prostate cancer^26^. Pathway activity analysis predicted that the WNT pathway was more active in the luminal 4 subgroup, but all other pathways were predicted to be less active (Supplementary material, Fig. S8D).

Using a gene set derived from epithelial stem cells^27^, we also found that the luminal 4 subgroup had a significantly higher stem cell signature score compared to all other epithelial subgroups (*p* < 0.00001, Mann-Whitney U test), including the basal and club cell groups (Fig. 3E). To further characterize the 4 luminal subgroups, we performed a pseudotime analysis using all annotated epithelial cells. A UMAP was generated using the Leiden clustering method in Monocle3 for the epithelial cells and labeled using the annotation described above. Similar to a previous single-cell study of non-cancer prostate^18^, we assigned the cluster of basal cells as the starting point for cell ordering and pseudotime trajectory calculation. Pseudotime trajectory was then superimposed on the epithelial cell UMAP for visualization, which, according to the pseudotime trajectory, indicated that basal cells gave rise to club cells and to luminal cells. This trajectory was consistent with previous scRNA-seq analyses of prostate tissues^18,19^. Interestingly, within the luminal cells, we found that the luminal 4 subgroup gave rise to the other luminal subgroups, suggesting that it may represent a luminal progenitor cell state (Fig. 3F). A gene set signature for luminal 4 subgroup was also derived using the highly expressed genes and using this gene set we detected this luminal progenitor-like subgroup in a separate prostate cancer scRNA-seq dataset^19^ (Supplementary material, Fig. S9A). In this integrated prostate cancer dataset, the luminal 4 subgroup cluster contained both BPH and prostate cancer cells that highly expressed ribosomal genes such as *RPL12*, *RPL7A*, and *RPL13A* (Supplementary material, Fig. S9B). We also detected the luminal 4 subgroup in an integrated BPH and normal dataset^21^. The cells with the highest luminal 4 subgroup signature were found in the BPH cells, but were extremely rare and constituted 0.1% of the entire luminal population (Supplementary material, Fig. S9C). 

To investigate the interaction of these epithelial cells with the surrounding stromal population and fibroblasts specifically, we performed a ligand-receptor interaction analysis between the stromal populations (fibroblasts and pericytes) and three luminal subgroups (subgroup 1,2 and 4) using CellPhoneDB (Fig. 3G). We detected 25 significantly enriched interactions between the luminal 4 subgroup and interstitial or peri-epithelial fibroblasts (Supplementary material, Table S4). 4 of these 25 interactions featured macrophage migration inhibitory factor (MIF).

MIF is a macrophage-associated proinflammatory factor that is overexpressed in prostate cancers^28^. *MIF* was found to be highly expressed in the luminal 4 subgroup, compared to the other luminal groups (Supplementary material, Fig. S7) and the complex formed with its main receptor, *CD74*, was predicted to be a significant interaction between the luminal 4 and the interstitial and peri-epithelial fibroblast subgroups. Epidermal growth factor receptor (*EGFR*) to *MIF* was also predicted to be a significant interaction between both fibroblast groups and this progenitor-like luminal 4 subgroup alone but not the other two luminal subgroups. Between both fibroblast groups and progenitor-like luminal cells, *MIF* interacted with *TNFRSF14* and *TNFRSF10D* (Supplementary material, Fig. S10), which are cell surface receptors and members of the tumor necrosis factor receptor superfamily that drives inflammation and immune response^29^. These results suggest that a luminal subgroup may harbor interactions with fibroblasts that are associated with inflammatory pathways and could contribute to BPH.

### Myeloid cell subgrouping analysis reveals 4 distinct inflammation-related macrophage subtypes

To further investigate how signaling from the luminal 4 subgroup might regulate inflammation, we first performed a detailed annotation of immune cells according to their differentially expressed genes via unsupervised clustering (Supplementary material, Fig. S11A). Lymphoid (*IL7R*+) cells were predominant and T cells comprised the largest portion of the lymphoid groups, particularly the CD4+ (*n* = 1,177) and CD8+ (*n* = 1,728) subgroups. Using marker genes from Tuong et al.^30^, we identified CD4 tissue resident memory (TRM) cells (*n* = 465), CD16 + natural killer cells (*n* = 276), CD16- natural killer cells (*n* = 219), neutrophils (*n* = 892), and B cells (*n* = 363). By selecting the optimal resolution for clustering, we were also able to identify rare populations such as plasmacytoid dendritic cells (*n* = 51) and mast cells (*n* = 115). All subgroups had contributions from 14 to 15 patients (supplementary material, Figure S11A), except for neutrophils, which had contributions from 9 patients.

**Fig. 4:**
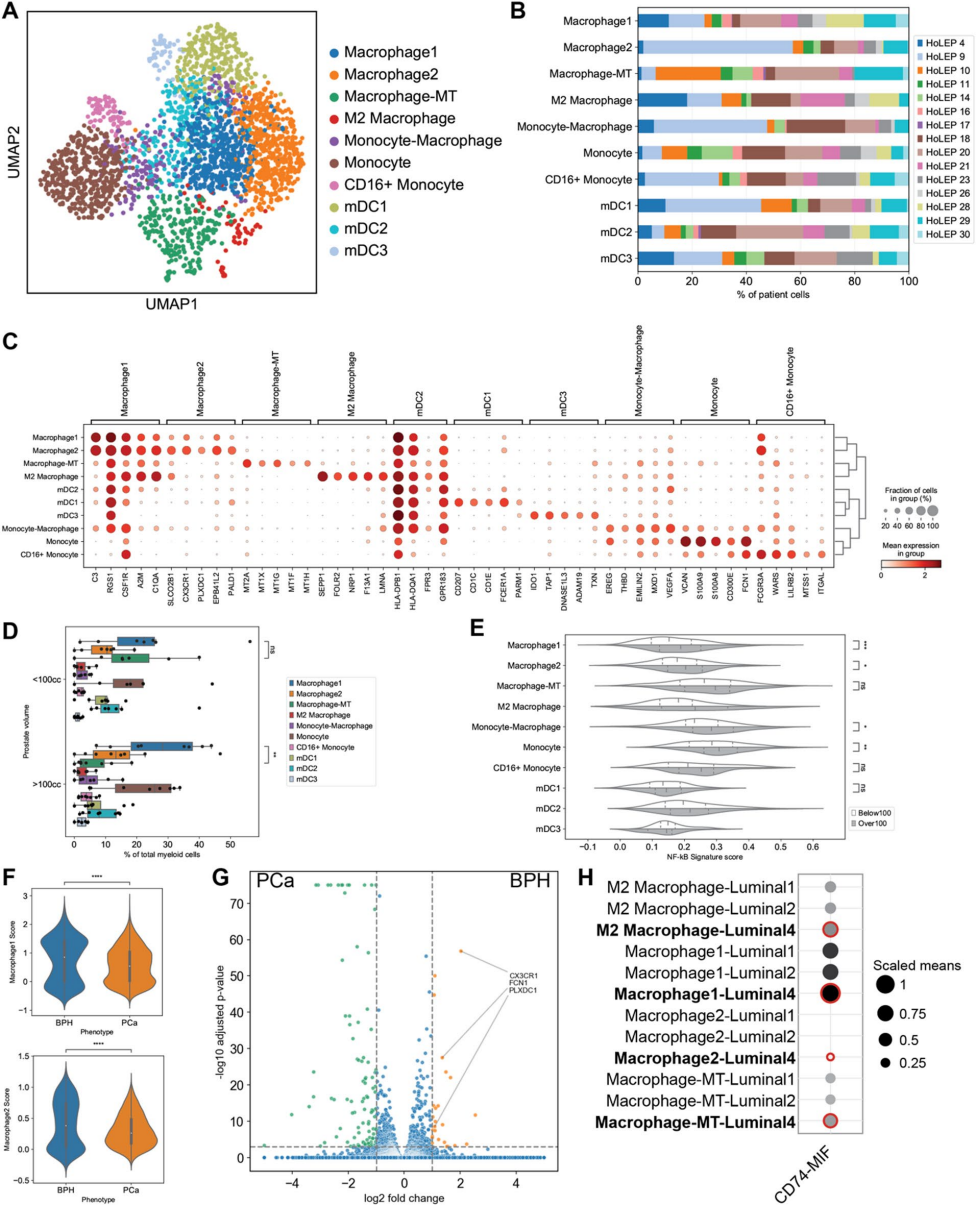
Myeloid characterization reveals 4 distinct macrophage subgroups. (**A**) UMAP shows clustering of myeloid subgroups. (**B**) Patient contribution to each myeloid cell subgroup. (**C**) Dot plot of marker gene expression in the 10 myeloid subgroups. (**D**) Myeloid subtype composition from all patients or stratified by prostate size (cc). (**E**) Violin plot of NF-kB pathway gene expression score across the 10 myeloid subgroups. (**F**) Violin plot of Macrophage1 and Macrophage2 signature scores in macrophages from BPH and prostate cancer datasets. (**G**) Volcano plot of differentially expressed genes from BPH (orange) and prostate cancer (green) macrophages. (**H**) Interaction analysis of luminal subgroups and macrophages. Larger dot sizes represent higher means of average expression level of the two interacting molecules. Red highlights represent a significant interaction, with an adjusted p-value < 0.05.

Myeloid lineage cells (*CD14*+) were the second most abundant group of immune cells and were further subclustered into 10 subgroups. (Fig. 4A). All patients were found to have each of these cell subgroups (Fig. 4B). Four separate macrophage groups (Macrophage1, Macrophage2, Macrophage-MT, and M2 Macrophage) were identified using differential gene expression analysis (Fig. 4C, Supplementary material, Table S5). The Macrophage1 group strongly expressed *C3*^31^ (*n* = 517), while Macrophage2 strongly expressed *CX3CR1*^32^; *C3* and *CX3CR1* are known inflammation regulators in macrophages and were found in all patients. The Macrophage2 group also expressed *PLXDC1* and *PALD1* (*n* = 500), both previously noted as inflammatory markers^33,34^. The third group, Macrophage-MT expressed multiple metallothionein genes, such as *MT1X*^35^ and *MT2A*^30^ (*n* = 229), which have been found in other datasets^18^ and are associated with changes in prostate tumor microenvironments. The three macrophage groups all strongly expressed genes associated with the M1 inflammatory phenotype such as *IL1B* and *PTGS2* (Supplementary material, Fig. S11C). We also detected a small population of M2 macrophages (*n* = 55), characterized by strong expression of *SEPP1*, *FOLR2*, *NRP1*, and *F13A1*. We identified a base monocyte group (*n* = 429), a CD16+ (*n* = 77) monocyte groups, and a transitional monocyte-macrophage group (*n* = 153). While the base monocyte group strongly expressed *CD14* and *CD36*, suggesting these may be identified as classical monocytes, both the base and CD16 + monocyte groups shared expression of genes associated with both classical and nonclassical monocytes (Supplementary material, Fig. 10D). We further annotated multiple dendritic cell subgroups. The base dendritic cell group expressed multiple HLA genes and *FCER1A* (*n* = 215), while another group additionally expressed *CD207* and *CD1C* (*n* = 266), both known dendritic cell surface markers. The third smaller group of dendritic cells (*n* = 45) expressed *IDO1* and *DNASE1L3*, two genes associated with the tumor microenvironment. Prostates with volumes larger than 100 cc also contained a greater ratio of Macrophage1 to Macrophage-MT macrophages (Figs. 4D, S11B). Genes from the inflammatory NF-κB pathway were highly expressed in macrophages and monocytes from these patients with larger prostates (Fig. 4E). This suggests that patients with more severe BPH have higher levels of inflammation. Using these specific markers for the four macrophage groups, we found all three macrophage populations in a separate prostate cancer dataset^19^ (Supplementary material, Fig. S12A). The Macrophage1 and Macrophage2 populations constitutes a significantly larger share of macrophages in BPH compared to prostate cancer (*p* < 0.00001, Mann-Whitney U test) (Fig. 4F). *CX3CR1*, *FCN1*,* and PLXDC1* were also found to be significantly upregulated in BPH macrophages compared to prostate cancer macrophages (Fig. 4G), indicating that the two more abundant macrophage groups may be primarily contributing to the BPH inflammation phenotype.

Ligand-receptor interaction analysis between the epithelial and immune cells revealed that only MIF-associated interactions were significantly enriched between all four macrophage groups and the progenitor-like luminal 4 group compared to the other luminal groups. The Macrophage1-Luminal4 interaction harbored the highest mean expression of the gene pair CD74-to-MIF (Fig. 4H, Supplementary material, Table S6). We found additional unique interactions, MIF-TNFRSF14 and MIF-TNFRSF10D, predicted to occur between the progenitor-like luminal subgroup (luminal 4) and two macrophage populations (Supplementary material, Fig. S12B). These three ligand-receptor pairs were also predicted to be statistically significant between monocytes and progenitor-like luminal subgroup (luminal 4) (Supplementary material, Fig. S12C), compared to monocytes and the other luminal subgroup. These findings indicate that macrophages may be activated or recruited by MIF secreted from the luminal 4 subgroup, further suggesting that the BPH inflammation phenotype could be linked to this interaction.

## Discussion

To our knowledge, our study is the first scRNA-seq analysis of BPH cells collected by HoLEP. With HoLEP becoming the standard for surgical treatment of BPH^36^, samples generated by the procedure can reveal biological insights about the origins of diseases in the prostate. Here we used single cell transcriptomic profiling to characterize samples extracted through HoLEP and found multiple stromal, epithelial, and immune subgroups that were linked to inflammation. Peri-epithelial fibroblasts expressing *CCL2* in our study had the highest expression of NF-κB pathway genes compared to all other stromal cells and may be associated with the sustained cell recruitment and inflammation found in BPH stromal cell layers. CCL2 is a chemoattractant that attracts both myeloid^37^ and lymphoid cells under different contexts^38^ and is involved in psoriasis^39^, atherosclerosis^40^, and other inflammation-related diseases^37^. Interestingly, *CCL2* is not typically expressed in normal prostate fibroblasts, but is a biomarker for prostate tumor microenvironments^41^. Increased *CCL2* expression has been associated with the proliferation of prostate stromal cells^42^ and is highly expressed in BPH fibroblasts. It has also been implicated as a potential factor in prostate cancer cell migration and infiltration^43^. The Macrophage1 and Macrophage2 subgroups in this cohort also have strong inflammatory profiles and are driven by known immunomodulatory genes associated with classical M1 macrophages. In particular, *CX3CR1* expressed by the two most abundant macrophages has been reported as a potential drug target for inflammatory diseases like psoriasis^44^ and atherosclerosis^45^.

Ligand-receptor interaction analysis predicted unique interactions involving MIF between the progenitor-like luminal subgroup and fibroblast, macrophage, and monocyte subgroups. MIF is a pro-inflammatory cytokine that plays a regulatory role in both the innate and adaptive immune responses^46^. Some studies have shown that MIF can promote tumor growth and other diseases^47,48^. With regard to the prostate, luminal epithelial cells express MIF and MIF expression is significantly higher in BPH epithelium compared to normal epithelium^49^. Modulating MIF overexpression may also lead to better clinical outcomes for BPH patients^50^. MIF can also affect signaling cascades through its binding to CD74. CD74 is heavily involved with cell survival and proliferation^51^ and this complex activates the mitogen-activated protein kinase (MAPK) and phosphoinositide 3-kinase (PI3K) pathways associated with proliferation and angiogenesis^51^. MIF signaling can also increase the expression of the inflammatory cytokines IL-6 and IL-8^52^ and CD74 directly interacts with CXC-chemokine receptor 2 (CXCR2) to form a receptor complex for IL-8 that can potentially result in an inflammatory feedback loop^53^. Our current study predicted CD74-MIF as a unique interaction between multiple cell types and the potential progenitor luminal subgroup, suggesting that this progenitor-like luminal subgroup may contribute to the BPH phenotype. In particular, the *CCL2* + peri-epithelial fibroblasts and *CX3CR1+ * Macrophage1 macrophages were shown to have strong interactions with the progenitor-like luminal cells through this complex, potentially demonstrating how these luminal cells drive inflammation through fibroblast and macrophage activation.

The progenitor-like luminal subgroup may represent a novel epithelial cell state or subtype associated with uncontrolled growth and proliferation. Compared to the other luminal subgroups, these cells highly express *KLK4*, which has been characterized as a potential biomarker for prostate, breast, and ovarian cancers^54–56^. The regulation of *KLK4* by androgens^57^ and its function as a proliferative factor in the development of prostate cancer^58^ suggest a possible role for this *KLK4*-high luminal subgroup in the promotion of prostatic enlargement. In addition, gene ontology analysis revealed a significant enrichment of established ribosomal gene sets in these *KLK4*-high cells. As ribosomal biogenesis is a crucial biological process closely tied to cell growth and proliferation^59^, this luminal subgroup could be associated with the development of prostatic hyperplasia.

Currently, alpha-adrenergic blockers and 5-ARIs are used to treat BPH^60,61^. While alpha-blockers minimize lower urinary tract symptoms by inhibiting smooth muscle contraction, 5-ARIs reduce prostate volume by decreasing dihydrotestosterone production. Although 5-ARI has been shown to improve clinical outcomes successfully, not all patients respond and some develop resistance^62^. Recent data indicate that atrophy of prostate luminal cells caused by 5-ARI correlated with reduced AR signaling and increased NF-κB signaling. 5-ARI also induced a luminal-to-club cell transition^63^. In our study, while the inflammatory TN/NF-κB pathway was higher in fibroblasts from patients with larger prostates, fibroblasts from patients treated with 5-ARIs had lower expression of these inflammation-associated genes. Across all fibroblast groups, fibroblasts from patients that were previously treated with 5-ARIs were lower in abundance than untreated patients.

Our results suggest the MIF-CD74 interaction as a potential target in BPH patients, particularly ones that exhibit high inflammation, and may benefit from next-generation MIF targeting drug development. Targeted MIF inhibitors, such as CPSI-1306^64^, benzoxazole-2-thione^65^, and *N*-acetyl-*p*-benzoquinone imine^66^, have demonstrated the ability to disrupt the binding of MIF to CD74. These inhibitors have shown some promise in clinical trials for cancer and autoimmune disease^49,50,52^ and may be potential therapeutic options for BPH.

Due to the increasing popularity of HoLEP as a treatment method for BPH, our study demonstrates that analyses on cells collected from this procedure can further contribute to our understanding of BPH. Our analyses identified a luminal epithelial subgroup as a potential contributor to the inflammation phenotype associated with BPH through *MIF* expression and its interactions with stromal and immune cells. While further studies are needed to investigate these interactions as pathways and targets for BPH, our results show that single cell analyses can identify unique cell types and interactions associated with BPH.

## Methods

### Experimental details

#### Sample collection

Fresh prostate tissue was obtained from fifteen patients undergoing holmium laser enucleation of the prostate (HoLEP) at the University of California San Francisco (UCSF) between January 2021 and January 2022. Patient demographics, comorbidities, and clinical data, including prostate size, and the presence or absence of 5-α reductase inhibitor usage, were obtained (Table 1). All specimens were confirmed pathologically to be non-cancerous in nature.

#### Study approval

The UCSF Institutional Review Board (IRB) committee approved the collection of the patient data included in this study. All relevant ethical regulations for work with human participants were compiled, and written informed consent was obtained.

#### Tissue collection and dissociation

Patients were positioned in the lithotomy position. The 2 lateral lobes and median lobe of the prostate were enucleated using a Holmium laser^67^. Each lobe was dissected away from the surgical capsule in a distal to proximal approach and released from the bladder neck. Prostate tissue was minced and evacuated by a Piranha morcellator (Richard Wolf). Morcellation was performed according to manufacturer’s recommendations up to 1,500 rpm with dual inflow saline irrigation. Morcellated tissue was collected in a canister and placed on ice prior to dissociation and preparation for single cell RNA-sequencing. Morcellated tissue was immediately delivered to the laboratory and further minced to ~ 1-5mm^3^ pieces with surgical scissors in a petri dish with 10 mL RP-10 (RPMI + 10% FBS) on ice for up to 5 min and washed with cold RP-10 (RPMI + 10% FBS). Each sample was centrifuged at 1,200 rpm x5 mins at 4 °C and resuspended in 10 mL warmed digestive media (HBSS + 1% HEPES) with 1,000 U/mL collagenase type IV (Worthington, Cat: LS004188), and rotated for 30 min at 37 °C. Samples were triturated by pipetting 10 times at the end of the incubation. Each sample was filtered through a 70-µm filter (Falcon, Cat: 352350), washed with RP-10, centrifuged at 1,200 rpm x5 mins at 4 °C, washed again with RP-10, and resuspended in RP-10. A hemocytometer was used to count the cells^68,69^.

#### Single-cell RNA sequencing

Sequencing was largely based on the Seq-Well S^3 protocol^23,24^. One to two arrays were used per sample. Each array was loaded as previously described with approximately 110,000 barcoded mRNA capture beads (ChemGenes, Cat: MACOSKO-2011-10(V+)) and with 15,000–20,000 cells. Arrays were sealed with functionalized polycarbonate membranes (Sterlitech, Cat: PCT00162 × 22100) and were incubated at 37 °C for 40 min.

After sealing, each array was incubated in lysis buffer (5 M guanidine thiocyanate, 1 mM EDTA, 0.5% sarkosyl, 1% BME). After detachment and removal of the top slides, arrays were rotated at 50 rpm for 20 min. Each array was washed with hybridization buffer (2 M NaCl, 4% PEG8000) and was then rocked in a hybridization buffer for 40 min. Beads from different arrays were collected separately. Each array was washed ten times with wash buffer (2 M NaCl, 3 mM MgCl_2_, 20 mM Tris-HCl pH 8.0, 4% PEG8000) and scraped ten times with a glass slide to collect beads into a conical tube.

For each array, beads were washed with Maxima RT buffer (ThermoFisher, Cat: EP0753) and resuspended in reverse transcription mastermix with Maxima RT buffer, PEG8000, Template Switch Oligo, dNTPs (NEB, Cat: N0447L), RNase inhibitor (Life Technologies, Cat: AM2696) and Maxima H Minus Reverse Transcriptase (ThermoFisher, Cat: EP0753) in water. Samples were rotated end-to-end, first at room temperature for 30 min and then at 52 °C overnight. Beads were washed once with TE-TW, once with TE-SDS, twice with TE-TW, and once with 10 mM Tris-HCl pH 8.0. They were treated with exonuclease I (NEB), rotating for 50 min at 37 °C. Beads were washed once with TE-SDS and twice with TE-TW. They were resuspended in 0.1 M NaOH and rotated for 5 min at room temperature. They were subsequently washed with TE-TW and TE. They were taken through second strand synthesis with Maxima RT buffer, PEG8000, dNTPs, dN-SMRT oligo, and Klenow Exo- (NEB, Cat: M0212L) in water. After rotating at 37 °C for 1 h, beads were washed twice with TE-TW, once with TE and once with water.

KAPA HiFi Hotstart Readymix PCR Kit (Kapa Biosystems, Cat: KK2602) and SMART PCR Primer (Supplementary material, Fig. S1) were used in whole transcriptome amplification (WTA). For each array, beads were distributed among 24 PCR reactions. Following WTA, three pools of eight reactions were made and were then purified using SPRI beads (Beckman Coulter), first at 0.6× and then at a 1× volumetric ratio.

For each sample, one pool was run on an HSD5000 tape (Agilent, Cat: 5067–5592). The concentration of DNA for each of the three pools was measured via the Qubit dsDNA HS Assay kit (ThermoFisher, Cat: Q33230). Libraries were prepared for each pool, using 1000 pg of DNA and the Nextera XT DNA Library Preparation Kit. They were dual-indexed with N700 and N500 oligonucleotides.

Library products were purified using SPRI beads, first at 0.6× and then at a 1× volumetric ratio. Libraries were then run on an HSD1000 tape (Agilent, Cat: 50675584) to determine the concentration between 400 and 800 bp. For each library, 3 nM dilutions were prepared. These dilutions were pooled for sequencing on a NovaSeq S4 flow cell.

The sequenced data were preprocessed and aligned using the dropseq_workflow on Terra (app.terra.bio). A digital gene expression matrix was generated for each sample, parsed, and analyzed following a customized pipeline.

#### Histology and immunohistochemistry

FFPE prostate tissue banked during HoLEP surgery was sliced to 4 μm and mounted on positively charged Superfrost microscope slides. Hematoxylin and eosin (H&E) staining was performed using a standard method. KRT5 (Abcam ab52635, 1:500) and KLK4 (Sigma hpa051839, 1:100) immunohistochemistry (IHC) was performed on serial sections at a 1:500 dilution after a 10-minute pH 6.0 citrate antigen retrieval at 100 °C on a Leica Bond III platform.

### Quantification and statistical analysis

#### Alignment and QC

Sequencing results were received as paired FASTQ files. Each pair of FASTQ files was aligned against GRCh38 reference genome using a WDL-based Dropseq workflow (https://cumulus.readthedocs.io/en/latest/drop_seq.html) on Terra. All default parameters were used in the alignment except the parameter –dropseq_tools_force_cells set as 2,000 to ensure maximum cell capture. The alignment results include aligned and corrected bam files, gene expression matrices in .txt.gz format, and txt files of alignment reports such as the list of barcodes, the number of reads, and alignment summaries. For each sample, the median and average number of genes captured per barcode were 419 and 628. The median and average number of UMIs captured per barcode were 818 and 1,511. The average percentage of mitochondrial content per barcode was 23.5%. Each gene expression matrix was then processed through decontX^70^ for ambient RNA-decontamination. Barcodes with less than 300 genes, 500 UMIs, or more than 20% of mitochondrial content were removed from downstream analysis. A total number of 16,234 cells were analyzed with an average of 981 genes, 1,601 UMIs and 5.3% of mitochondrial content (Supplementary material, Fig. S2).

#### Clustering analysis

Single-cell cluster analysis was performed with custom scripts utilizing the Seurat package (v4.3.0) in R (v4.2.0) and Scanpy package (v1.7.2) in Python (v3.6.8). Data from the samples were merged, as integration of the data did not discernibly alter cell clustering analysis. Individual cell UMI-collapsed read count matrices were loaded for analysis. We removed 2,850 genes that were detected in 3 or less cells and all mitochondrial genes from downstream analyses. Highly variable genes with a minimum and maximum gene expression of > = 0.0125 and < = 3, respectively and a minimum dispersion of genes = 0.5 were selected. Gene expression of all cells was normalized by multiplying by a scale factor of 10,000 and subsequently log-transformed. Using 50 calculated principal components and the Leiden algorithm, we identified 26 clusters. Marker genes were defined by a Wilcoxon pairwise differential expression analysis in Scanpy. Feature and violin plots were used to visualize gene expression.

#### Differential gene analysis

Differential gene analysis was performed using the rank_genes_group module in Scanpy, which performs a Wilcoxon rank sum test between groups.

#### Pseudotime analysis

To assess the epithelial cell states regarding their orders in the differentiation trajectory, we conducted pseudotime analysis on all epithelial cells in the HoLEP scRNA-seq dataset using Monocle3 (https://cole-trapnell-lab.github.io/monocle3/). The epithelial cell object was processed and converted to a cds object for Monocle3 compatibility. This cds object was then preprocessed with principal component analysis (PCA) in Monocle3, followed by Leiden clustering. A graph representation was computed using Uniform Manifold Approximation and Projection (UMAP) and epithelial annotations were projected on this UMAP visualization. Based on established studies, we selected basal cells as the starting point and the cells were ordered for the pseudotime trajectory calculation. Specific gene expression levels along the trajectory were also visualized.

#### Gene ontology and gene set enrichment analysis

The package GSEAPY (v1.0.4) was used to predict differences in pathway enrichment between subgroups or phenotypes. Gene sets from MSigDB served as references for these analyses. The package decoupleR (v1.6.0) was also used to predict differences in pathway activity.

#### Ligand receptor analysis

The package CellPhoneDB (v3.1.0) was used to predict significant ligand-receptor interactions between different cell types. Cell subgroups were downsampled to 200 cells prior to analysis. Significant mean and cell communication significance (*p* < 0.05) was calculated for cell subgroups and patients.

## Electronic supplementary material

Below is the link to the electronic supplementary material.

Supplementary Material 1

Supplementary Material 2

Supplementary Material 3

## Data Availability

The raw single cell RNA-sequencing data and differential gene expression matrix files generated during this study are available in the Gene Expression Omnibus (GEO) under the accession number GSE290213. Two previously published scRNA-seq datasets were analyzed and used to confirm annotation of the cell types - GSE176031 and GSE117403. The datasets generated by and analyzed in this study are also available by request from FWH.
