# Supplementary material for: A single cell transcriptional profile of benign prostatic hyperplasia
Source: Sci Rep. 2026 Mar 14;16:9556. doi: 10.1038/s41598-025-02417-w (PMC13009279; doi:10.1038/s41598-025-02417-w)
Supplement: Supplementary file 1 — Supplementary Material 1 [file 41598_2025_2417_MOESM1_ESM.docx]

| ***Primer*** | ***Sequence*** |
| --- | --- |
| *TSO* | *AAGCAGTGGTATCAACGCAGAGTGAATrGrGrG* |
| *dN-SMRT* | *AAGCAGTGGTATCAACGCAGAGTGANNNGGNNNB* |
| *SMART PCR Primer* | *AAGCAGTGGTATCAACGCAGAGT* |
| *Custom Read 1 Primer* | *GCCTGTCCGCGGAAGCAGTGGTATCAACGCAGAGTAC* |

###### Figure S1 - SMART PCR Primer

Primer sequences used for whole transcriptome amplification.


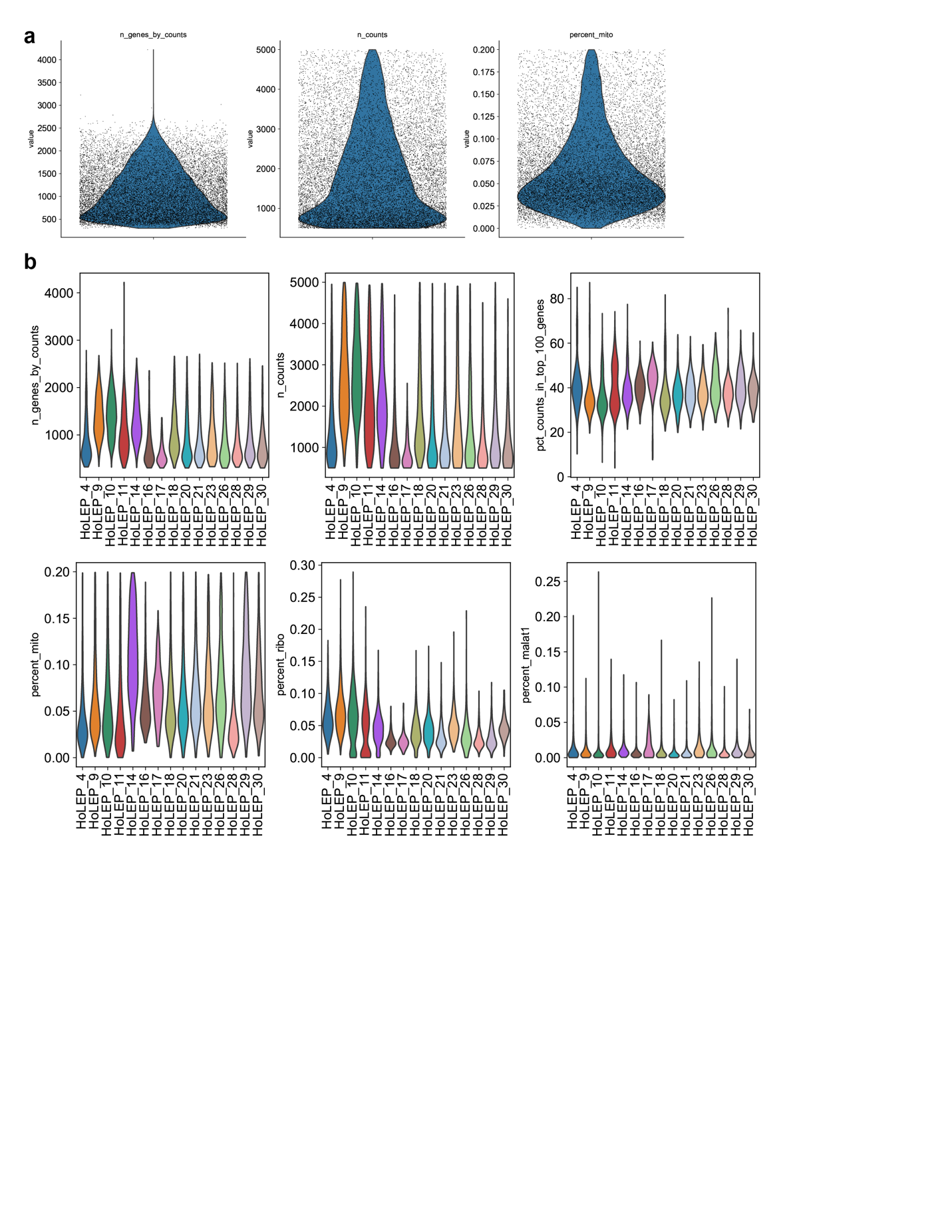


###### Figure S2 – Quality metrics

(A) Violin plots of genes, UMIs, and ribosomal gene content across the entire dataset. (B) Violin plots of genes, counts, cumulative percentage of counts for the 100 most expressed genes in a cell, ribosomal proportion of total counts for a cell, mitochondrial proportion of total counts for a cell, and *MALAT1* proportion of total counts for a cell by sample.


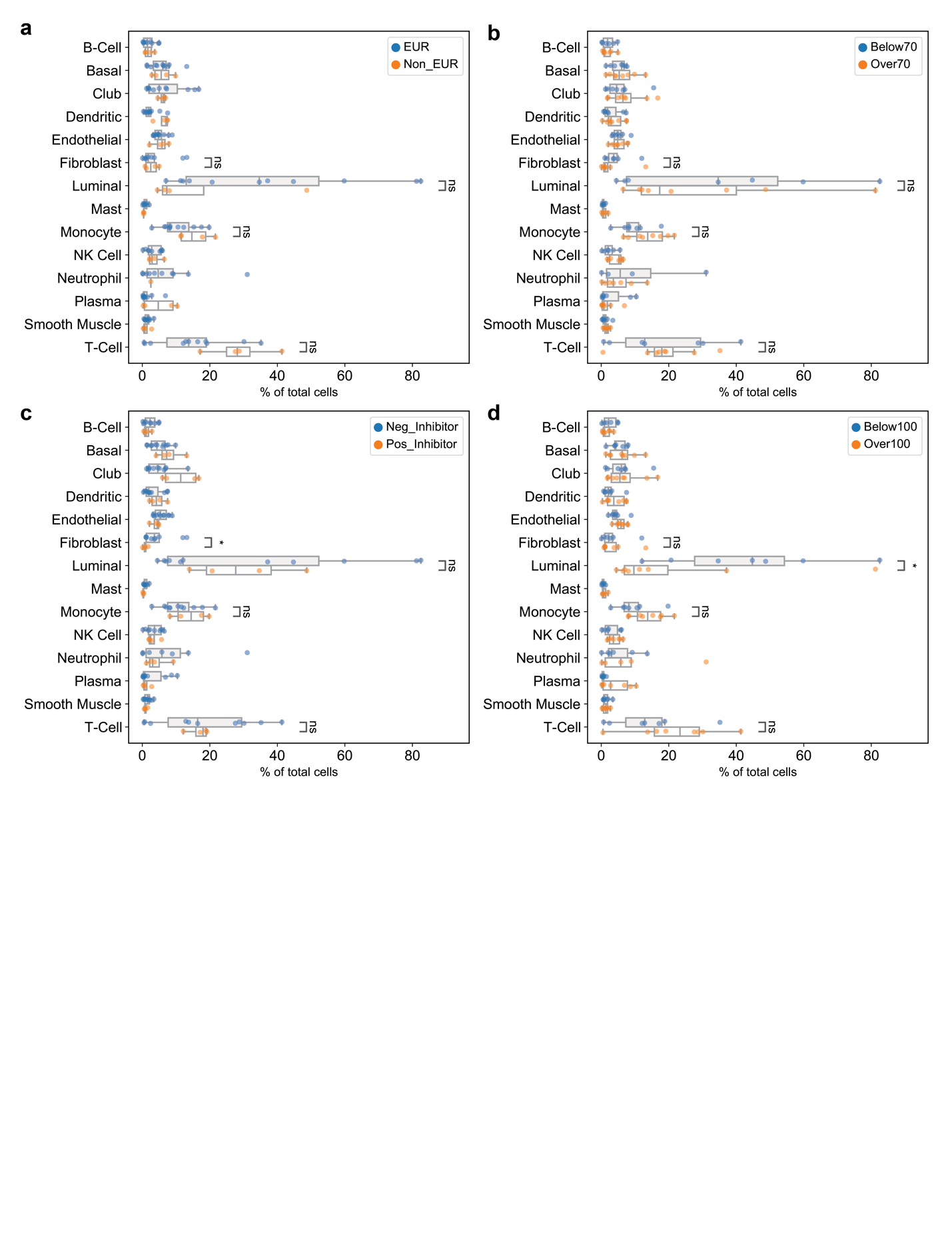


###### Figure S3 - Cell number ratios based on patient clinical features

(A) Boxplots of cell number ratios from broad cell types based on ethnicity. (B) Boxplots of cell number ratios from broad cell types based on patient age. (C) Boxplots of cell number ratios from broad cell types based on 5-ARI treatment (p < 0.05, Mann-Whitney U test). (D) Boxplots of cell number ratios from broad cell types based on prostate size (p < 0.05, Mann-Whitney U test).


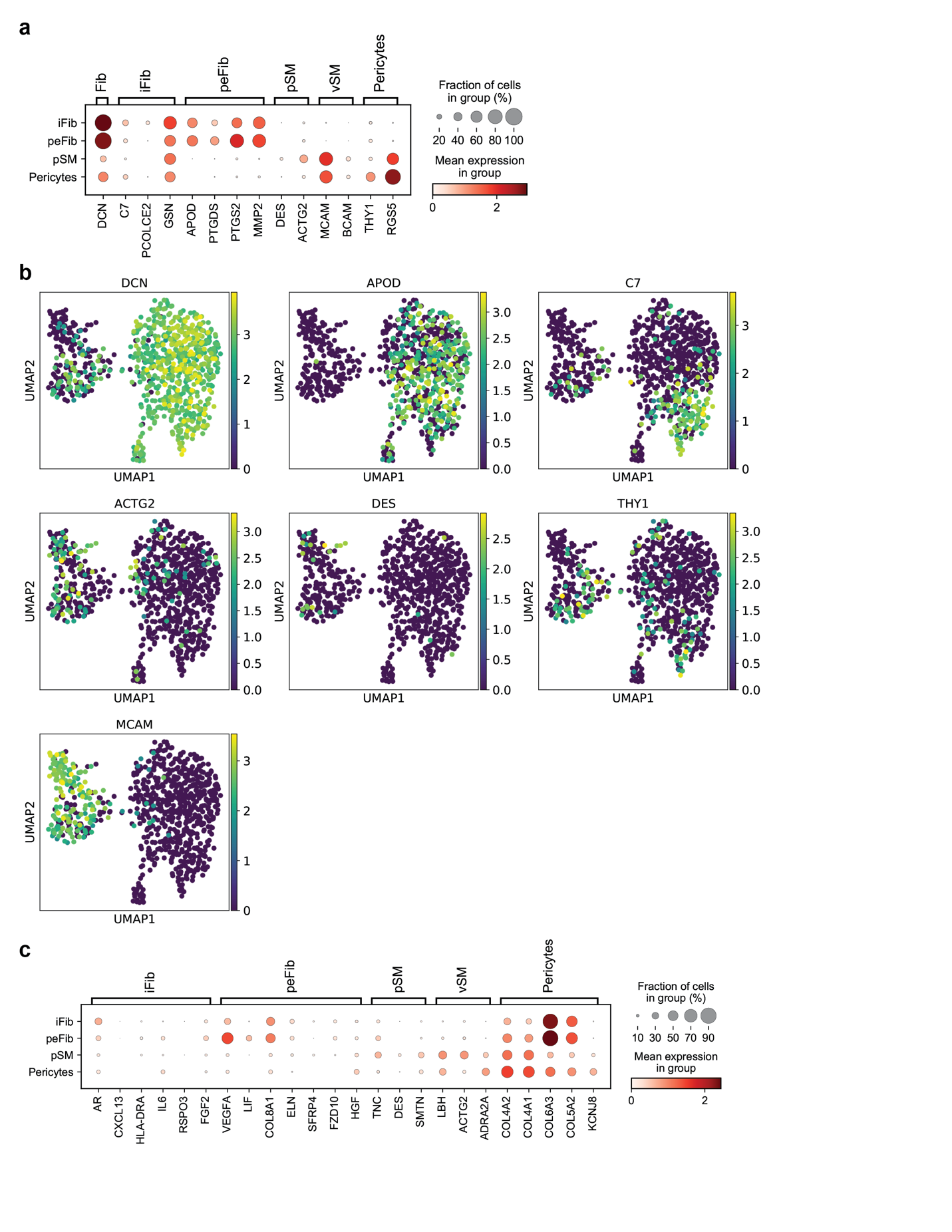


###### Figure S4 - Stromal cell marker comparison

(A) Dot plot of stromal marker genes highly expressed in normal prostate samples from Joseph et al., 2021. (B) Feature plots of previously described marker genes. (C) Dot plot of stromal marker genes highly expressed in BPH samples from Joseph et al., 2021.


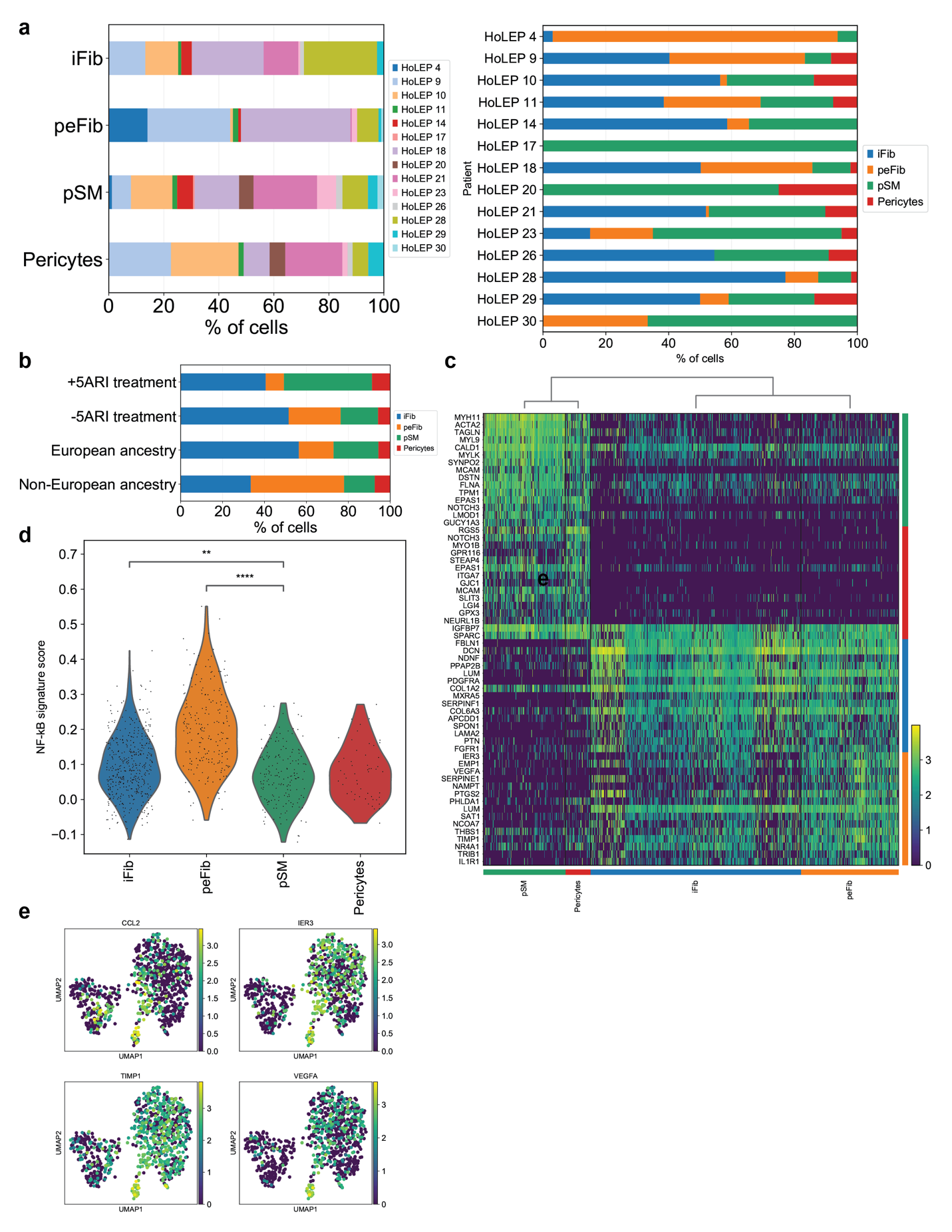


###### Figure S5 - Stromal cell characterization

(A) Cell contributions from each patient. (B) Cell number ratios of stromal cells in patients based on 5-ARI treatment or ancestry. (C) Heatmap of differentially expressed genes from each stromal cell group. (D) Signature score derived from NF-κB pathway associated genes of each stromal cell group. (E) Feature plots of *CCL2*, *IER3*, *TIMP1*, and *VEGFA* expression.


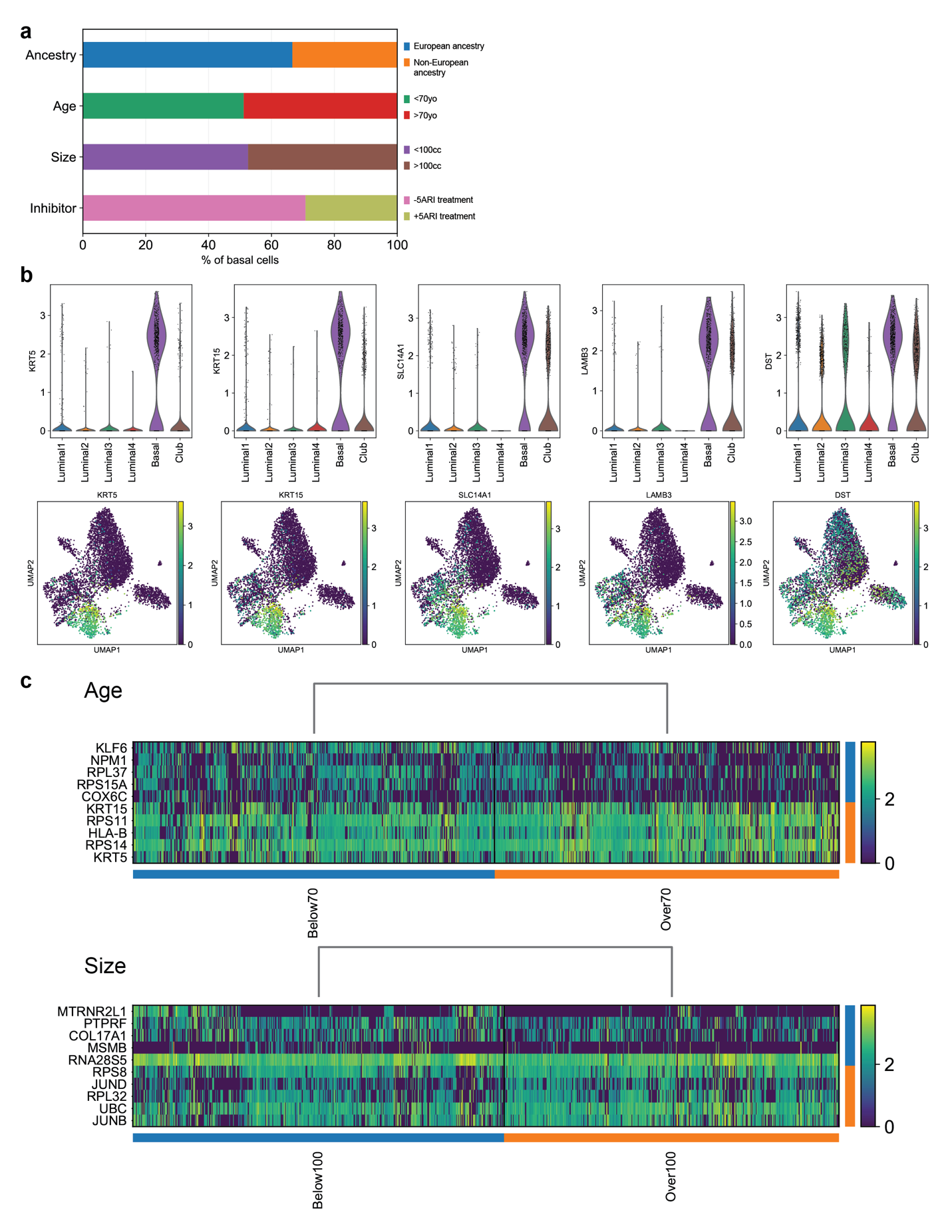


###### Figure S6 - Basal cell marker genes

(A) Basal cell counts across ancestry, patient age, prostate size, and 5ARI treatment status. (B) Violin plots and feature plots of marker gene expression in basal cells. (C) Heatmaps of differentially expressed genes in basal cells, divided by patient age or prostate size.


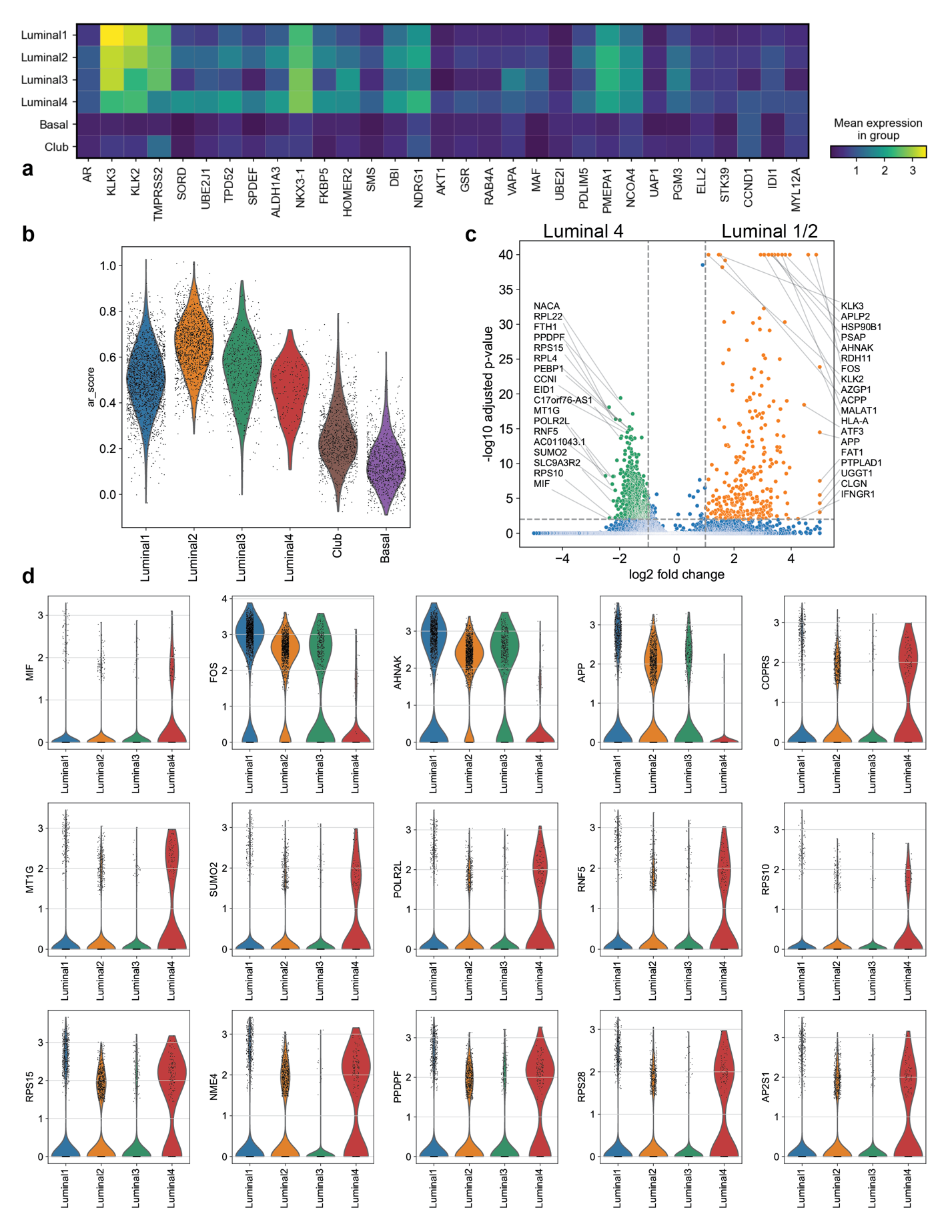


###### Figure S7 - Luminal cell marker and AR pathway gene expression

(A) Matrix plot of AR pathway gene expression in the 6 epithelial subgroups. (B) Violin plots of AR pathway signature scores in the 6 epithelial subgroups. (C) Volcano plot of differentially expressed genes found in the luminal 4 subgroup (green) or combined luminal 1 and 2 subgroups (orange). (D) Violin plots of marker gene expression in the 4 luminal subgroups.


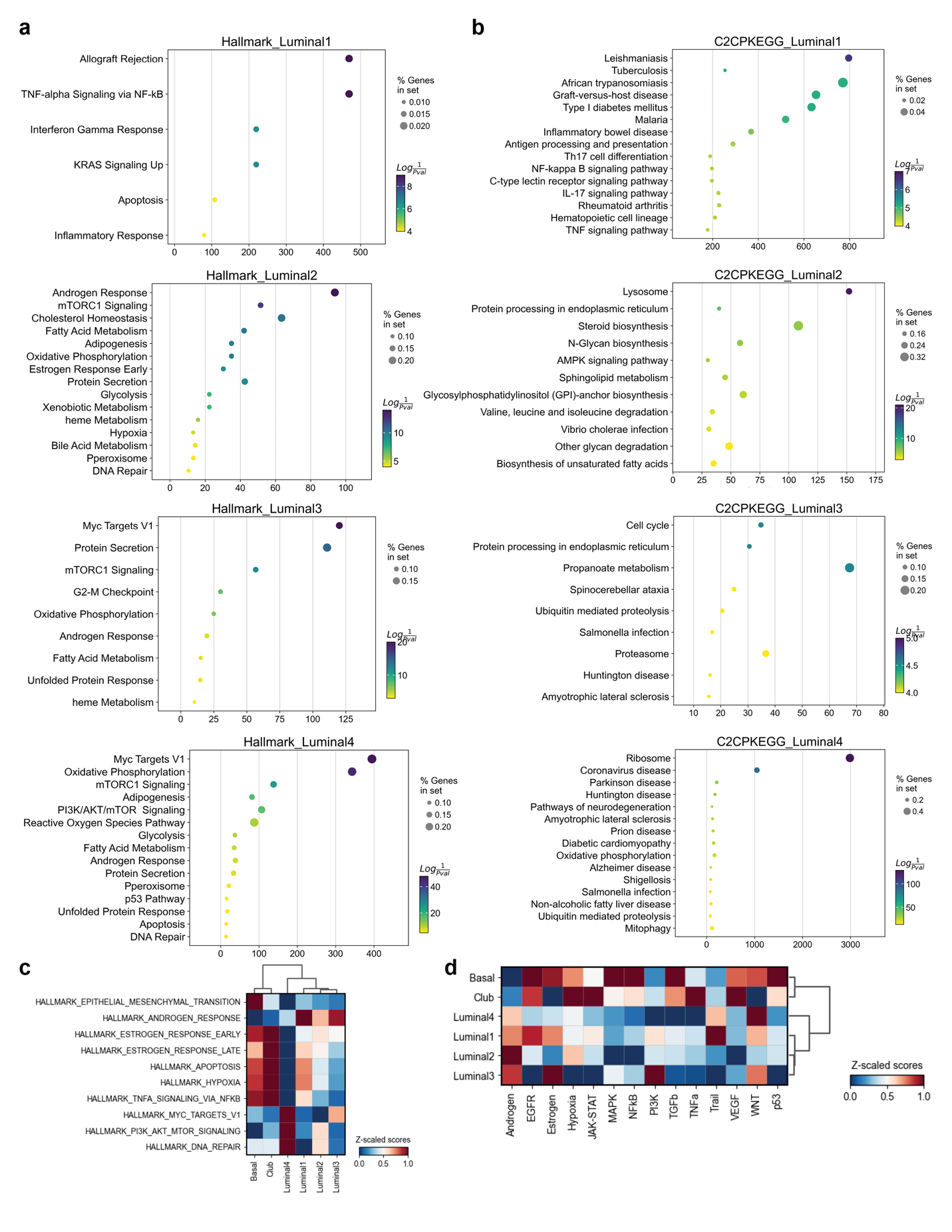


###### Figure S8 - Gene set enrichment analysis of epithelial subgroups

(A) Gene set enrichment analysis of differentially expressed genes from the 4 luminal subgroups. Genes were compared to Hallmark gene sets. (B) Gene set enrichment analysis of differentially expressed genes from the luminal subgroups. Genes were compared to gene sets from canonical pathways (CP) and Kyoto Encyclopedia of Genes and Genomes (KEGG). (C) Gene set enrichment analysis of selected Hallmark gene sets from the epithelial subgroups. (D) Pathway activity analysis of epithelial subgroups.


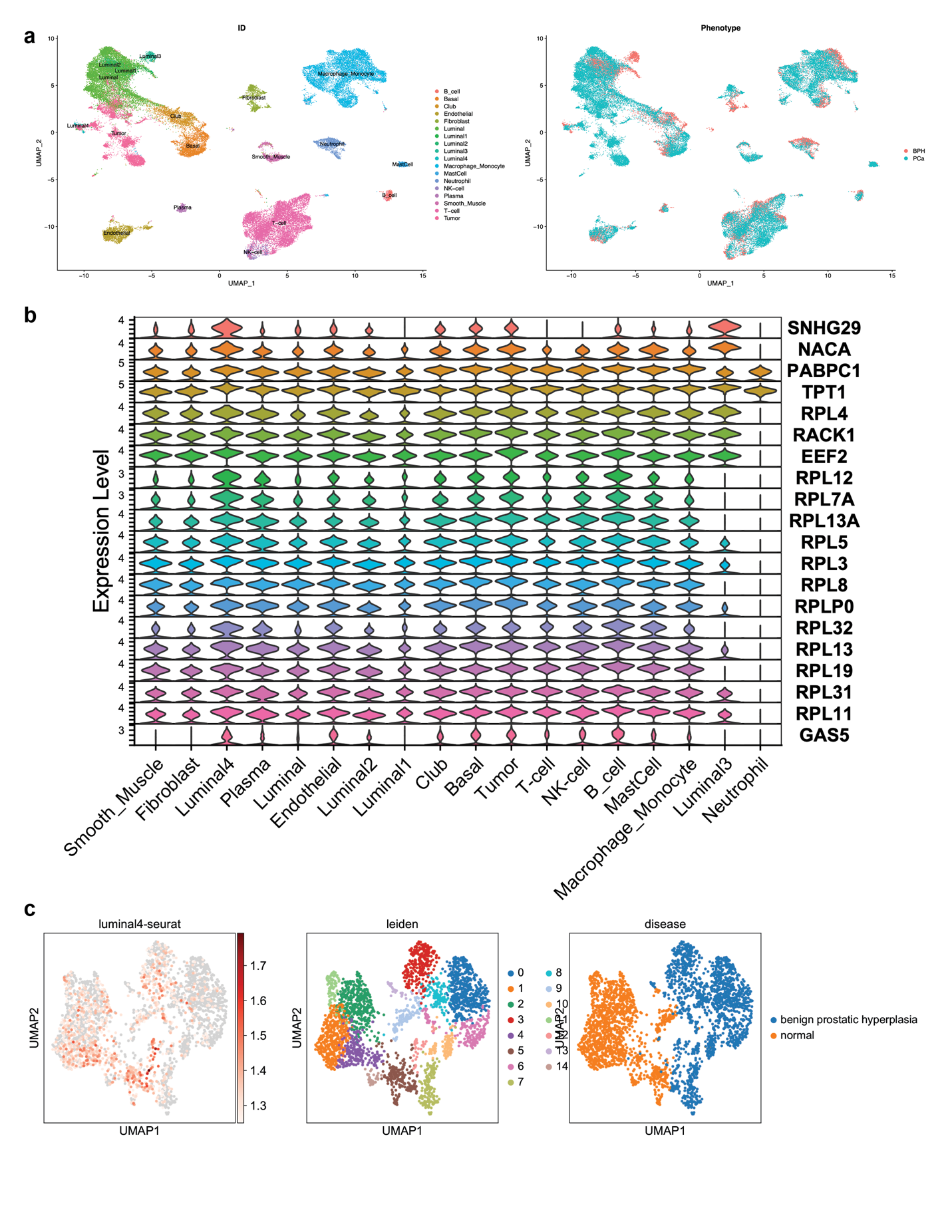


###### Figure S9 - Detection of the luminal 4 subgroup in a prostate cancer dataset

(A) UMAP shows clustering of the integrated BPH and prostate cancer datasets. Cells were grouped by either by cell annotation or dataset. (B) Gene expression of the luminal 4 marker genes in the integrated dataset. (C) UMAPs of integrated BPH and normal prostate datasets. Marker genes from the luminal 4 subgroup were used to score the integrated dataset.


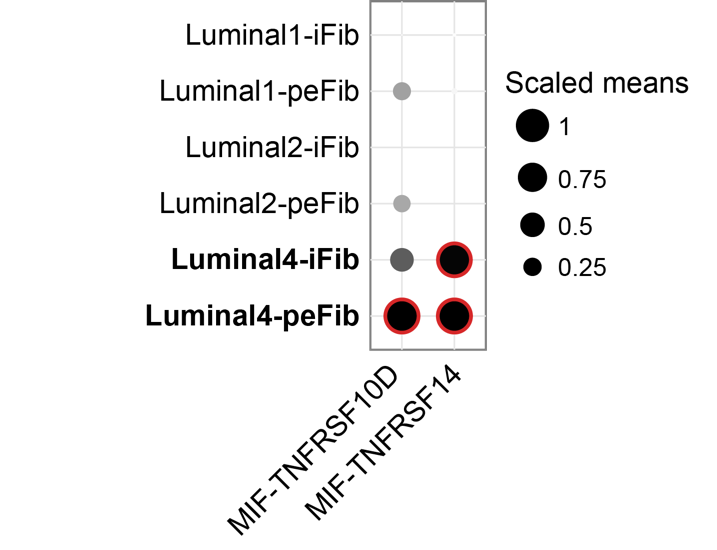


###### Figure S10 – Luminal-stromal Interaction analysis

(A) Interaction analysis of the three luminal subgroups and stromal cells. Larger dot sizes represent higher means of average expression level of the two interacting molecules. Red highlights represent a significant interaction, with an adjusted p-value <0.05.


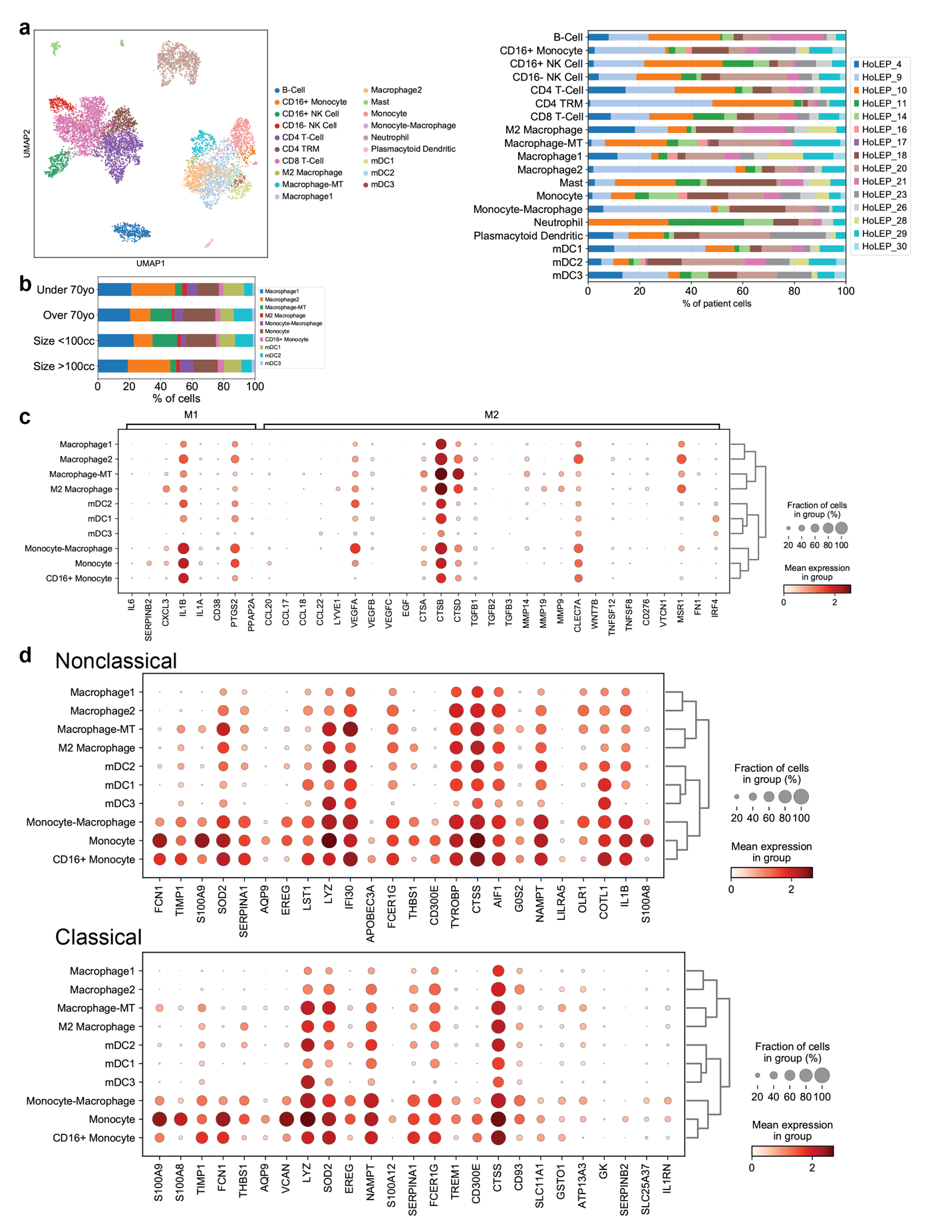


###### Figure S11 - Immune cell characterization

(A) UMAP of all immune subtypes and patient contribution to each immune cell type. (B) Total immune cell composition based on patient age or prostate size. (C) Dot plot of M1 and M2 macrophage marker gene expression in the 10 myeloid subgroups. (D) Dot plot of nonclassical (above) and classical (below) monocyte marker gene expression in the 10 myeloid subgroups.


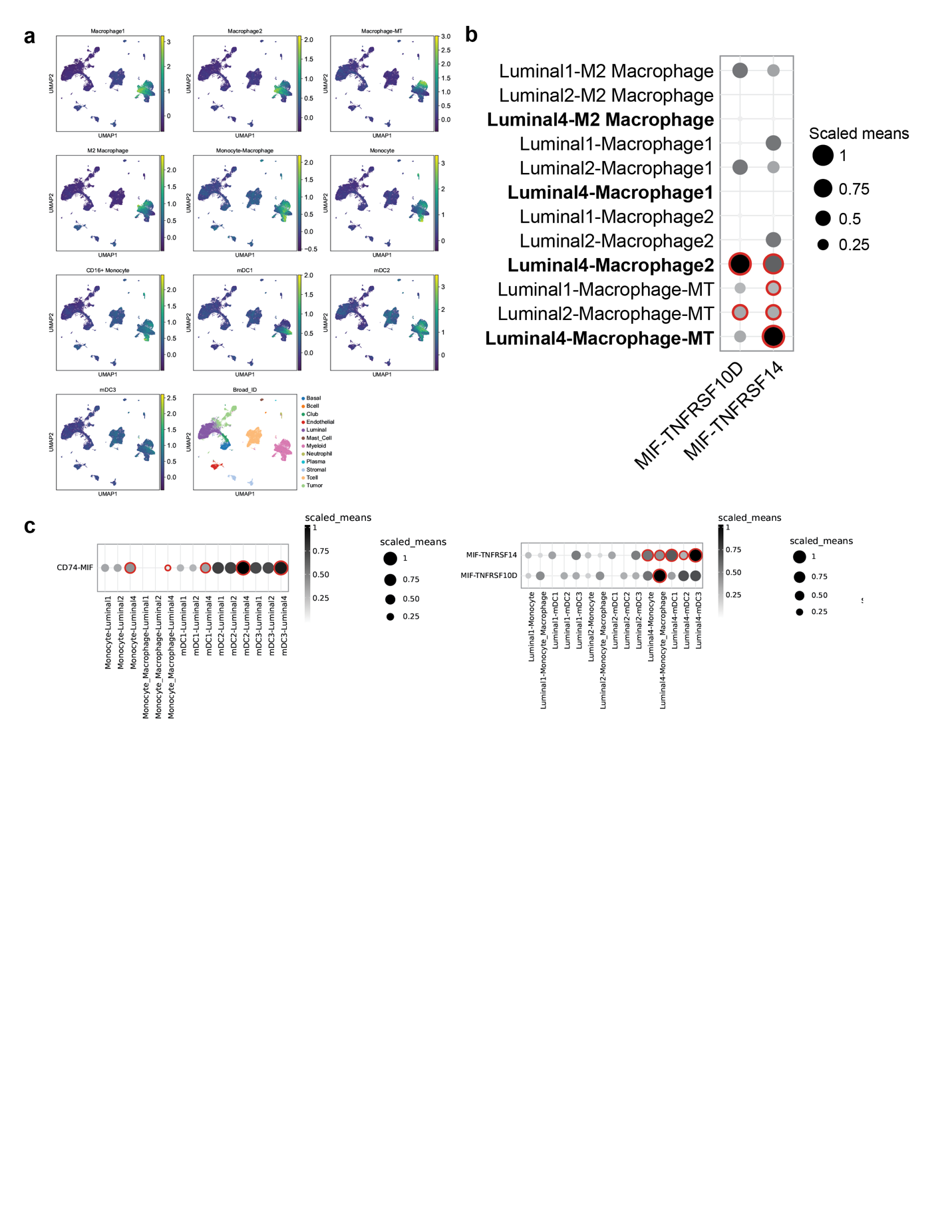


###### Figure S12 – BPH myeloid cells in external dataset and myeloid-luminal4 interaction analysis

(A) UMAPs of external prostate cancer dataset. Cells were scored based on marker genes from each myeloid subgroup. (B) Interaction analysis of the luminal 4 subgroup and macrophages. Larger dot sizes represent higher means of average expression level of the two interacting molecules. Red highlights represent a significant interaction, with an adjusted p-value <0.05. (C) Interaction analysis of the luminal 4 subgroup and other myeloid cells. Larger dot sizes represent higher means of average expression level of the two interacting molecules. Red highlights represent a significant interaction, with an adjusted p-value <0.05.
