# Supplementary material for: A single cell transcriptional profile of benign prostatic hyperplasia
Source: Sci Rep. 2026 Mar 14;16:9556. doi: 10.1038/s41598-025-02417-w (PMC13009279; doi:10.1038/s41598-025-02417-w)
Supplement: Supplementary file 2 — Supplementary Material 2 [file 41598_2025_2417_MOESM2_ESM.docx]

###### Table S1 – Gene signatures for 14 clusters

Marker genes for each cluster were defined by Wilcoxon pairwise differential expression analysis. Z-scores were calculated for each gene in each cluster.

###### Table S2 – Cell type composition of 15 BPH samples

Cell type by percent of the 14 clusters for each sample.

###### Table S3 – Gene signatures for 4 stromal subgroups

Marker genes for each cluster were defined by Wilcoxon pairwise differential expression analysis. Z-scores were calculated for each gene in each cluster.

###### Table S4 – Ligand-receptor interaction analysis from CellPhoneDB between luminal and stromal subgroups

Interaction analysis between luminal and stromal subgroups was performed with CellPhoneDB. Mean expression of the gene participants in the interaction are listed for each interaction pair.

###### Table S5 – Gene signatures for 10 myeloid subgroups

Marker genes for each cluster were defined by Wilcoxon pairwise differential expression analysis. Z-scores were calculated for each gene in each cluster.

###### Table S6 – Ligand-receptor interaction analysis from CellPhoneDB between luminal and myeloid subgroups

nteraction analysis between luminal and stromal subgroups was performed with CellPhoneDB. Mean expression of the gene participants in the interaction are listed for each interaction pair.
